# Supplementary material for: Interactive SARS-CoV-2 mutation timemaps
Source: F1000Res. 2021 Jun 3;10:68. Originally published 2021 Feb 3. [Version 2] doi: 10.12688/f1000research.50857.2 (PMC8188262; doi:10.12688/f1000research.50857.2)
Supplement: Peer review history

# Peer review: Reviewer response for version 2

**DOI:** 10.5256/f1000research.57062.r86694

**Published:** 2021-06-21

**Authors:** Ingo Ebersberger, Ruben Iruegas

**Affiliations:** 1 Applied Bioinformatics Group, Institute for Cell Biology and Neuroscience, Goethe-University Frankfurt, Frankfurt, Germany

I have no further comments.

Is the work clearly and accurately presented and does it cite the current literature?

Partly

If applicable, is the statistical analysis and its interpretation appropriate?

Not applicable

Are all the source data underlying the results available to ensure full reproducibility?

Yes

Is the study design appropriate and is the work technically sound?

Partly

Are the conclusions drawn adequately supported by the results?

Partly

Are sufficient details of methods and analysis provided to allow replication by others?

Yes

Reviewer Expertise:

BioSequence-Informatics

We confirm that we have read this submission and believe that we have an appropriate level of expertise to confirm that it is of an acceptable scientific standard.

# Peer review: Reviewer response for version 2

**DOI:** 10.5256/f1000research.57062.r86693

**Published:** 2021-06-14

**Authors:** Jale Moradi

**Affiliations:** 1 Department of Microbiology, Faculty of Medicine, Kermanshah University of Medical Sciences, Kermanshah, Iran

The new version of the manuscript is improved in all sections, more details are provided in the result part by some examples that show the utility of the maps. The results indicate that the script using to generate maps is changed, since, the interactive function of the maps has improved and it is possible to zoom on each spot. I believe this interactive evolutionary vector graphic map could be generated and used by other researchers in different projects to monitor the evolution of the SARS-CoV-2 virus.

Is the work clearly and accurately presented and does it cite the current literature?

Yes

If applicable, is the statistical analysis and its interpretation appropriate?

Not applicable

Are all the source data underlying the results available to ensure full reproducibility?

Yes

Is the study design appropriate and is the work technically sound?

Partly

Are the conclusions drawn adequately supported by the results?

Partly

Are sufficient details of methods and analysis provided to allow replication by others?

Yes

Reviewer Expertise:

Medical Microbiology, Genomics, Microbial Evolution

I confirm that I have read this submission and believe that I have an appropriate level of expertise to confirm that it is of an acceptable scientific standard.

# Peer review: Reviewer response for version 2

**DOI:** 10.5256/f1000research.57062.r86695

**Published:** 2021-06-08

**Authors:** Takahiko Koyama

**Affiliations:** 1 TJ Watson Research Center, IBM, Scarsdale, NY, USA

The tool is now more interactive with zooming to look into more details. Although I personally do not understand utility of concentric representation, there is not much to add before my approval for the indexing.

Is the work clearly and accurately presented and does it cite the current literature?

Partly

If applicable, is the statistical analysis and its interpretation appropriate?

Not applicable

Are all the source data underlying the results available to ensure full reproducibility?

Yes

Is the study design appropriate and is the work technically sound?

Yes

Are the conclusions drawn adequately supported by the results?

Partly

Are sufficient details of methods and analysis provided to allow replication by others?

Yes

Reviewer Expertise:

Genomics, bioinformatics, oncology, immunology, virology, and stem cell biology.

I confirm that I have read this submission and believe that I have an appropriate level of expertise to confirm that it is of an acceptable scientific standard.

# Peer review: Reviewer response for version 1

**DOI:** 10.5256/f1000research.53946.r85512

**Published:** 2021-06-01

**Authors:** Jale Moradi

**Affiliations:** 1 Department of Microbiology, Faculty of Medicine, Kermanshah University of Medical Sciences, Kermanshah, Iran

The authors have geographically shown the nucleotide variations for global SARS-CoV-2 sequences in a time map. The sequences have been downloaded, polished and analyzed with ntHit and ntEdit. The Wuhan-Hu-1-NC_045512/MN908947 was set as the reference sequence, then the variations output was mapped based on the sample collection time by a script written in PERL. The results have shown in two circle maps including “whole viral genome” and “spike protein gene” variations over time from January 1 ^st^ 2020 as day 1 to December 31 ^st^ 2020 as day 366. Each radius in these circles represents a day and each spot on this radius shows a variation. Also, the spots are shown in different colours that each colour is indicating a specific geographical region (continent or country).

 It is a useful tool to overview the evolution of the virus since the beginning of the epidemic. Furthermore, it can be concluded which part of the genome has more variations, also, the colour appearance of the map helps us to understand approximately how many mutations there are in different regions or from which ones the mutations originated. If it were possible to identify the relevant mutation (exact mutation type) by clicking on each spot, it could help more. Also, different spots have overlaps in many parts, which would provide better information if it was possible to determine which spots this overlap includes.

 Overall, the developed script provides a useful map for viewing the pattern of virus evolution globally, although it would be more informative if the authors could improve this script to solve the mentioned issues.

Is the work clearly and accurately presented and does it cite the current literature?

Yes

If applicable, is the statistical analysis and its interpretation appropriate?

Not applicable

Are all the source data underlying the results available to ensure full reproducibility?

Yes

Is the study design appropriate and is the work technically sound?

Partly

Are the conclusions drawn adequately supported by the results?

Partly

Are sufficient details of methods and analysis provided to allow replication by others?

Yes

Reviewer Expertise:

Medical Microbiology, genomics, immunology

I confirm that I have read this submission and believe that I have an appropriate level of expertise to confirm that it is of an acceptable scientific standard, however I have significant reservations, as outlined above.

# Response

**Published:** 2021-06-03

**Authors:** René Warren

**Affiliations:** Canada's Michael Smith Genome Sciences Centre, Canada

We thank our Reviewer for their support of our work and insights. We also value the suggestions, as it helps us improve upon the work and broaden the interest.

 We have just published a revised version of the manuscript (v2), which expands on the utility of the maps, situates them in context of other similar work, and introduces new map features to increase interactivity and overall experience.

 Some of the maps' new features (since original submission):

Interactivity

1. Maps are draggable.
2. Zoom/pan.
3. Tilt 90 degrees to make axis horizontal (this and above features implemented in a navigation wheel).
4. Colour highlight on mutation tooltip.
5. Gene/variant views have additional colour highlight (by region) on certain maps*.

*The added functionality comes at a cost, making them sluggish when views are too dense, which is why this feature is currently only used to display individual genes/variant displays and not the whole genome

Improvements:

1. Over 120 individual displays, all SARS-CoV-2 genes are now presented.
2. Better discrimination of close high-frequency mutations allows more information to show through by adjusting the spot ratio (r=sqrt(freq*factor/pi) and no longer plots on a log10 in ratio mode.
3. When same %, adjust a secondary sort such that the colour matches the first region labelled.
4. Better grouping/sorting of overlapping points.
5. Added ability to explore switch year from the current view 2020<->2021 and between ratio(%) and raw (#) counts without having to go to main menu and use drop-down.

The mutation "spots" are also plotted incrementally (by coordinates) and by decreasing order of frequency, allowing most mutations to interactively show (and not be obscured by overlaps). But overlaps are unavoidable with displays that are too dense, and some data points may still be out of reach, but other individual maps (eg. variant/gene levels) may provide a better visual of the most important mutations.

 Improvements 2), 3) and 4) in particular are in response to our Reviewer's comment on spot overlap, and calculating the ratio in such a fashion (instead of log10) enables a better resolution on close-by high-frequency mutations (such as the D614G). Most displays will show missense mutation to minimize display density, but we also offer representations by types (missense vs silent) and all-encompassing. With tooltip, the mutation type is shown as either its effect in amino acid space (eg. N501Y) or silent when the nucleotide variation has no predicted effect.

# Peer review: Reviewer response for version 1

**DOI:** 10.5256/f1000research.53946.r85294

**Published:** 2021-05-17

**Authors:** Takahiko Koyama

**Affiliations:** 1 TJ Watson Research Center, IBM, Scarsdale, NY, USA

Authors have developed a web based visualization tool for longitudinal evolution of SARS-CoV-2 genomes.

 Although they have made unique representation of longitudinal strain developments, it is not clear the utility of the tool. For instance, while concentric circle representation of daily genomes is visually appealing, it limits the duration to a year and inner part inevitably becomes crowded compared with outer area.

 Lack of interactivity is also an issue. There must have been a way to magnify the area.

 Furthermore, in mutation prone loci, the dots are overlapped and not easy to see what is going on. For these reasons, utility of the tool is limited; more improvements need to be done before it gains large user base.

Is the work clearly and accurately presented and does it cite the current literature?

Partly

If applicable, is the statistical analysis and its interpretation appropriate?

Not applicable

Are all the source data underlying the results available to ensure full reproducibility?

Yes

Is the study design appropriate and is the work technically sound?

Yes

Are the conclusions drawn adequately supported by the results?

Partly

Are sufficient details of methods and analysis provided to allow replication by others?

Yes

Reviewer Expertise:

Genomics, bioinformatics, oncology, immunology, virology, and stem cell biology.

I confirm that I have read this submission and believe that I have an appropriate level of expertise to confirm that it is of an acceptable scientific standard, however I have significant reservations, as outlined above.

# Response

**Published:** 2021-05-17

**Authors:** René Warren

**Affiliations:** Canada's Michael Smith Genome Sciences Centre, Canada

*Authors have developed a web based visualization tool for longitudinal evolution of SARS-CoV-2 genomes.*

*Although they have made unique representation of longitudinal strain developments, it is not clear the utility of the tool. For instance, while concentric circle representation of daily genomes is visually appealing, it limits the duration to a year and inner part inevitably becomes crowded compared with outer area.*

**We thank our Reviewer for the valuable insights provided and spending the time to review our work. We acknowledge limitations of the display, and we stress that our original work on this was done in December, on 200,000 GISAID genomes and one year's worth of data. Our preprint became public January 2021 and we subsequently submitted this work to F1000Research, summarizing the 2020 pandemic-associated SARS-CoV-2 variants for year 2020. A circular representation is an aesthetic choice, allowing to get a bird's eye view of the breadth of mutations.**

*Lack of interactivity is also an issue. There must have been a way to magnify the area.*

**This is a great suggestion. We have now added the ability to pan and zoom on each map, making the maps more interactive.**

*Furthermore, in mutation prone loci, the dots are overlapped and not easy to see what is going on. For these reasons, utility of the tool is limited; more improvements need to be done before it gains large user base.*

**The maps were first built to visually quantify the appreciable variability that exists in rapidly evolving SARS-CoV-2 genomes. Since, we have added spike-specific views, and variants of concerns (VOCs) to the list of maps available to the community. We also provide the tools to generate the maps, such that advanced users may customize and generate additional views of interest, as needed**

# Response

**Published:** 2021-06-03

**Authors:** René Warren

**Affiliations:** Canada's Michael Smith Genome Sciences Centre, Canada

We wanted to add to our previous response to our Reviewer. Once again, we are grateful for your suggestions to improve upon interactivity of the maps. Since your Review, we have worked to improve the user experience and we list below some of the new features:

Interactivity

1. Maps are draggable.
2. Zoom/pan.
3. Tilt 90 degrees to make axis horizontal (this and above features implemented in a navigation wheel).
4. Colour highlight on mutation tooltip.
5. Gene/variant views have additional colour highlight (by region) on certain maps*.

*The added functionality comes at a cost, making them sluggish when views are too dense, which is why this feature is currently only used to display individual genes/variant displays and not the whole genome

Overall improvements

1. Over 120 individual displays, all SARS-CoV-2 genes are now presented.
2. Better discrimination of close high-frequency mutations allows more information to show through by adjusting the spot ratio (r=sqrt(freq*factor/pi) and no longer plots on a log10 in ratio mode.
3. When same %, adjust a secondary sort such that the colour matches the first region labelled.
4. Better grouping/sorting of overlapping points.
5. Added ability to explore switch year from the current view 2020<->2021 and between ratio(%) and raw (#) counts without having to go to main menu and use drop-down.

Thanks again for spending the time to review our work.

# Peer review: Reviewer response for version 1

**DOI:** 10.5256/f1000research.53946.r83795

**Published:** 2021-05-14

**Authors:** Ingo Ebersberger, Ruben Iruegas

**Affiliations:** 1 Applied Bioinformatics Group, Institute for Cell Biology and Neuroscience, Goethe-University Frankfurt, Frankfurt, Germany

The authors present interactive mutation time maps for SARS-CoV-2, which provide a highly resolving view of when, where and how frequent a particular mutation was detected in the sampled SARS-CoV-2 genome sequences provided via GISAID. The manuscript itself is rather short. It is briefly describing the methodological approach of how the mutations have been detected and mapped to the reference genome. The combined Results and Discussion section is equally concise and comprises a description of what is seen in the interactive maps together with few example observations that can be made with these maps. The Discussion section ends with the expression of the hope that the maps presented here “will help researchers in their exploration of SARS-CoV-2 mutations and their predicted effect over time.”

 Overall, the topic that is touched in this manuscript is highly relevant, as variations of SARS-CoV-2 is something that currently is and will be of major concern in the future. Here, the graphs present a very nice access to the information that is represented by the ever-increasing amount of viral genome sequences world-wide. The data presentation is appealing, and it allows to overview the general trends of SARS-CoV-2 evolution. However, we see considerable room for (essential) improvement.

 Major issues:

 The authors end the manuscript with the belief that the interactive maps will be of help for the research community working on SARS-CoV-2 variation. We miss two things here:

 First, it would be great if the authors show how the data provided by the maps can be used to indeed come up with new conclusions, in particular with respect to the ‘predicted effect over time’. For us, it is entirely unclear how such an analysis should be performed. Exploring the data, this is something that one nicely can do while looking at the plots, some clear signals, e.g. the fate of D614G, can also be extracted. But how to work with the data beyond this simple and straightforward ‘looking’ at the plots? Please, don’t get us wrong here, we consider looking at data a very important aspect of data analysis. Still, the sheer amount of information, which results in very dense plots with many overlapping data points, makes it, in our opinion, very hard to identify emerging variants that should be monitored right from the start. Just to give you an example: D614G is represented by a very prominent circle in the plots. What would be the authors approach to identify and monitor a novel variant, say at position 615 of the reference strain? By looking at the plots, we consider this almost impossible, since the signal will be entirely covered by the prominent mutation at position 614.

 The analysis is presented using the “ground-zero” strain as a reference. But is this still timely? Numerous variants have now frequencies that go far beyond that of the original nucleotide at a certain position, again, for example the D614G variant. This would allow to ‘purge’ the signal of very successful variants, helping to direct the focus on emerging variants.

 When it comes to the website itself, we see some room for improvement:

- First and foremost, we think the plots are overcrowded with information. Although it is nice to see a global overview of the data across the entire genome, 365 days, and 6 continents, it is impossible (at least for me) to explore this information other than randomly clicking individual data points, as we have outlined above. we think, this approach would benefit from providing the information in more digestible data fractions. Thus far, the user can choose to focus on the spike, but not on the other proteins. It would be helpful, just as a suggestion, to focus also on variants with a certain prevalence. But we are sure that the authors will have way better ideas than our proposals here, once they specify how a user should work with the plots and the data. Looking at https://nextstrain.org, which also provides a very nice overview of SARS-CoV-2 variation, may give some hints.
- It would be very convenient, if the interactive plots would be designed such that the user can toggle the information for display, instead of having to go back to the main menu and select a different display mode.
- Trend lines that show the prevalence of a certain variant in a certain region over time would help a lot and should be easy to implement.
- The orientation of where in a genome a certain variant exists is very hard. Although the vertical bars at 12 h in the circular plot should indicate in what ORF a variant is located, this is really hard to track across the full plot. In particular, because the bar-ORF assignment is not visible.
- Animation of daily variant emergence is again a nice feature. However, it is a gif and not interactive. The time lapse does not allow the user to pause, fast forward, or skip to a particular time. Moreover, x-axis labels overlap in particular for the spike. This makes the plot nice to look at, but the information that can be retrieved is only limited.
- Graph of weekly spike protein variant emergence is not interactive and difficult to read, as the lines overlap with each other and some have similar colors. Some functionalities could be implemented such as being able to toggle strains from the right menu, selecting a time range and continent/country, and being able to hover over to display the information. 2020 and 2021 plots have layout inconsistencies and could be merged into a single graph.
- The variant emergence graph heavily competes with the information in https://nextstrain.org, which claims to be updated daily.

In the outermost ring, we detected a variant that is assigned to na/na. What is this supposed to mean?

Is the work clearly and accurately presented and does it cite the current literature?

Partly

If applicable, is the statistical analysis and its interpretation appropriate?

Not applicable

Are all the source data underlying the results available to ensure full reproducibility?

Yes

Is the study design appropriate and is the work technically sound?

Partly

Are the conclusions drawn adequately supported by the results?

Partly

Are sufficient details of methods and analysis provided to allow replication by others?

Yes

Reviewer Expertise:

BioSequence-Informatics

We confirm that we have read this submission and believe that we have an appropriate level of expertise to confirm that it is of an acceptable scientific standard, however we have significant reservations, as outlined above.

# Response

**Published:** 2021-05-14

**Authors:** René Warren

**Affiliations:** Canada's Michael Smith Genome Sciences Centre, Canada

*The authors present interactive mutation time maps for SARS-CoV-2, which provide a highly resolving view of when, where and how frequent a particular mutation was detected in the sampled SARS-CoV-2 genome sequences provided via GISAID. The manuscript itself is rather short. It is briefly describing the methodological approach of how the mutations have been detected and mapped to the reference genome. The combined Results and Discussion section is equally concise and comprises a description of what is seen in the interactive maps together with few example observations that can be made with these maps. The Discussion section ends with the expression of the hope that the maps presented here “will help researchers in their exploration of SARS-CoV-2 mutations and their predicted effect over time.”*

*Overall, the topic that is touched in this manuscript is highly relevant, as variations of SARS-CoV-2 is something that currently is and will be of major concern in the future. Here, the graphs present a very nice access to the information that is represented by the ever-increasing amount of viral genome sequences world-wide. The data presentation is appealing, and it allows to overview the general trends of SARS-CoV-2 evolution. However, we see considerable room for (essential) improvement.*

**We thank our Reviewers for their comments, suggestions and diligence with their extensive report. Our response can be found below, in bold face**

*Major issues:*

*The authors end the manuscript with the belief that the interactive maps will be of help for the research community working on SARS-CoV-2 variation. We miss two things here:*

*First, it would be great if the authors show how the data provided by the maps can be used to indeed come up with new conclusions, in particular with respect to the ‘predicted effect over time’. For us, it is entirely unclear how such an analysis should be performed. Exploring the data, this is something that one nicely can do while looking at the plots, some clear signals, e.g. the fate of D614G, can also be extracted. But how to work with the data beyond this simple and straightforward ‘looking’ at the plots? Please, don’t get us wrong here, we consider looking at data a very important aspect of data analysis. Still, the sheer amount of information, which results in very dense plots with many overlapping data points, makes it, in our opinion, very hard to identify emerging variants that should be monitored right from the start. Just to give you an example: D614G is represented by a very prominent circle in the plots. What would be the authors approach to identify and monitor a novel variant, say at position 615 of the reference strain? By looking at the plots, we consider this almost impossible, since the signal will be entirely covered by the prominent mutation at position 614.*

**We greatly really appreciate community feedback on the potential usefulness of this work, and not only the maps, but additional analysis we were able to provide after we submitted the paper (our Reviewers made mentioned of them below), using the wealth of information we were able to mine from the GISAID genomes (these secondary analysis results, which consists of nucleotide variants and their effect, are tallied each week from each individual SARS-CoV-2 genome).  We originally built the maps to be fairly qualitative, to simply gain a [visual] appreciation for the rapid coronavirus evolution on a year scale/factoring sample regions of origin, and this is what we presented in the manuscript. In our conclusion we give an example of a mutation that is observable from the GISAID genomes, on our maps, at the time reported in published papers; Since submission, the GISAID catalogue has more than doubled in size and maps quickly became dense, as our Reviewer indicated. To help remedy the problem and make the maps more useful, we have since started to provide additional genome and spike views of variants of concerns (VOCs) and have added visualizations for 2021 (a more digestible data fraction, indicated below by our Reviewer). Another type of information that can be extracted from the maps is the speed at which mutations in VOCs have appeared and spreading in additional jurisdictions, which can be readily observed without too much effort. Our Reviewers are correct that variations in close proximity are difficult to see, which is why we provide views for the spike-encoding gene. Still, it would be difficult to differentiate between positions 614 and 615, which is why we provide the SVG-generating script such that interested parties would be able to generate custom views should they chose to (Ideally a more flexible website could help, see response below).**

*The analysis is presented using the “ground-zero” strain as a reference. But is this still timely? Numerous variants have now frequencies that go far beyond that of the original nucleotide at a certain position, again, for example the D614G variant. This would allow to ‘purge’ the signal of very successful variants, helping to direct the focus on emerging variants.*

**Our Reviewer is correct that the comparison is relative. When we started this project in December 2020, it made sense to use the "ground zero" strain genome. We could make the case for selecting another set of references to compare against, but it may lead to disagreements in scientific circles, on the base genome sequence to use. Additional maps may be produced in the future to see evolution within each VOCs, which may be an acceptable proposition.**

 When it comes to the website itself, we see some room for improvement:

- *First and foremost, we think the plots are overcrowded with information. Although it is nice to see a global overview of the data across the entire genome, 365 days, and 6 continents, it is impossible (at least for me) to explore this information other than randomly clicking individual data points, as we have outlined above. we think, this approach would benefit from providing the information in more digestible data fractions. Thus far, the user can choose to focus on the spike, but not on the other proteins. It would be helpful, just as a suggestion, to focus also on variants with a certain prevalence. But we are sure that the authors will have way better ideas than our proposals here, once they specify how a user should work with the plots and the data. Looking at https://nextstrain.org, which also provides a very nice overview of SARS-CoV-2 variation, may give some hints.*
- *It would be very convenient, if the interactive plots would be designed such that the user can toggle the information for display, instead of having to go back to the main menu and select a different display mode.*
- *Trend lines that show the prevalence of a certain variant in a certain region over time would help a lot and should be easy to implement.*
- *The orientation of where in a genome a certain variant exists is very hard. Although the vertical bars at 12 h in the circular plot should indicate in what ORF a variant is located, this is really hard to track across the full plot. In particular, because the bar-ORF assignment is not visible.*
- *Animation of daily variant emergence is again a nice feature. However, it is a gif and not interactive. The time lapse does not allow the user to pause, fast forward, or skip to a particular time. Moreover, x-axis labels overlap in particular for the spike. This makes the plot nice to look at, but the information that can be retrieved is only limited.*
- *Graph of weekly spike protein variant emergence is not interactive and difficult to read, as the lines overlap with each other and some have similar colors. Some functionalities could be implemented such as being able to toggle strains from the right menu, selecting a time range and continent/country, and being able to hover over to display the information. 2020 and 2021 plots have layout inconsistencies and could be merged into a single graph.*
- *The variant emergence graph heavily competes with the information in https://nextstrain.org, which claims to be updated daily.*

**We thank our Reviewers for spending the time to navigate the website, which originally, wasn't part of the project (built as a means to share the maps). We agree that a more modern and flexible web design would help with the customization and eventual uptake of these maps. Some of the plots were added to the website for convenience, to show users what is possible to do with the extensive mutation data we are compiling for this project (and available for download here)**

*In the outermost ring, we detected a variant that is assigned to na/na. What is this supposed to mean?*

**These variants fall in UTR regions. Thank you for the feedback, in our next release of the maps, we will replace NA by UTR to indicate that this nucleotide variant compared to the reference is found outside coding regions. The last position indicates possible effect in the protein space, which is not applicable in this case.**
